# Midwifery students’ experiences: Violations of dignity during childbirth

**DOI:** 10.1177/09697330231197703

**Published:** 2023-08-31

**Authors:** Arezoo Haseli, Shahla Khosravi, Saiedeh Sadat Hajimirzaie, Rozhin Feli, Dara Rasoal

**Affiliations:** 48464 Kermanshah University of Medical Sciences; Tehran University of Medical Sciences; 48478 Iran Ministry of Health and Medical Education; 48464 Kermanshah University of Medical Sciences; 101092 School of Health and Welfare, Dalarna University, Sweden

**Keywords:** Clinical ethics, topic areas, dignity in care < topic areas, midwifery, childbirth, qualitative study, student perspective

## Abstract

**Background:**

The principle of human dignity is woven into the ethical principles of the midwifery profession, noted as both an obligation and a human right.

**Research Objectives:**

The aim of this study is to explore the experiences of midwifery students regarding threats to women's dignity during childbirth.

**Research Design:**

This is a qualitative study with explorative design. Participants and Research Context: The research was carried out in 2022 at Kermanshah University of Medical Sciences, involving 32 midwifery students in individual interviews that lasted between 30 and 90 minutes. These participants aged 21 to 28 years, with an average age of 23.5 years, provided their perspectives on the matter.

**Results:**

Four key themes described the threats to women’s dignity during childbirth: 1) professional incompetence, 2) abuse of power imbalance, 3) caring only for physical and not mental health, and 4) structural issues within the healthcare system. Professional incompetence was characterized by outdated practices and lack of adherence to evidence-based medicine. Abuse of power imbalance was demonstrated in instances where the authoritative position of healthcare providers was misused, thereby disrupting the respectful care that women are entitled to receive. The disproportionate emphasis on physical health over mental health was evidenced by the disregard for mothers' psychological well-being during childbirth. Lastly, systemic structural issues emerged as significant impediments, revealing the need for system-wide changes. Ethical considerations: This study was approved by the Ethics Committee of the Research Deputy at Kermanshah University. Participation was voluntary and the confidentiality were maintained.

**Conclusion:**

The findings underscore the role that unprofessional behavior, ethical lapses in medical practices, and systemic challenges play in undermining maternal dignity during childbirth. These threats necessitate urgent attention and must be adequately addressed in policy development and program implementation to safeguard the dignity of mothers during childbirth.

## Introduction

Childbirth, as one of life's most impactful experiences, can have enduring physical and emotional effects.^1,2^ The fundamental importance of dignity and respect during labor, counterposed with the egregious violation of women's rights through obstetric violence, is globally acknowledged.^3,4^ This violation encompasses acts of disrespect, abuse, neglect, or harm throughout pregnancy, childbirth, or the postpartum period.^3,5–9^ Regrettably, numerous women worldwide encounter abuse rather than receiving the necessary care during childbirth.^3,7,10,11^

## Background

Violations against women can range from physical harm and unnecessary medical procedures to verbal abuse, neglect, or denial of pain relief. Obstetric violence intensifies the pain of childbirth and directly infringes upon a woman's dignity, contravening the fundamental principles of respect for an individual's autonomy, as well as physical and mental integrity.^6,8^

Dignity is a concept of significant relevance in the context of healthcare, where it plays a crucial role in shaping ethical principles,^12,13^ and guiding the provision of patient-centered care.^14^ In the healthcare setting, dignity encompasses the respect, value, and preservation of the inherent worth of every individual receiving care. Drawing upon the philosophical perspectives of Kant’s definition, dignity in healthcare can be understood as grounded in the rationality and autonomy of a person.^12,15^ Kant's emphasis on moral agency and rational decision-making aligns with the importance of respecting patients' autonomy^16^ and their right to make informed decisions about their own healthcare.^17^ Upholding dignity in healthcare requires addressing healthcare disparities, ensuring access to quality care, and promoting fairness in the distribution of healthcare resources.^12^ “To treat someone with dignity is to treat them as being of worth, in a way that is respectful of them as valued individuals.”^18^ Similarly, preserving the dignity of pregnant women through a holistic approach is a fundamental philosophy of the International Confederation of Midwives.^19^ Despite this, research indicates that many women around the world do not receive the necessary care during childbirth, instead encountering various forms of abuse.^7,11,20,21,22^

In healthcare, particularly in midwifery, student observations of women who has been treated with insufficient dignity, respect, and emotional support during childbirth are deeply concerning. These accounts reveal a significant gap in understanding the factors that can undermine women's dignity. This deficiency underscores the importance of enhancing the educational curriculum for midwifery students. Consequently, our study aims to explore midwifery students' firsthand experiences regarding threats to women's dignity during childbirth in their clinical practice. These insights can provide a deeper understanding of the specific hurdles and barriers students encounter in clinical practice that may compromise the dignity of the women they care for.

## Methods

This qualitative study, which used a conventional content analysis approach, was conducted from April to December 2022 at Kermanshah University of Medical Sciences in Kermanshah, Iran. The participants in this research were midwifery students who were in their 5th semester of bachelor's degree or higher and had experience in labor and delivery. We promoted the study within our institution, inviting students to participate voluntarily. We used purposeful sampling to ensure a diverse range of academic semesters among students from the Faculty of Nursing and Midwifery in Kermanshah.

The location for the study was determined based on mutual agreement between the researchers and participants, selecting a place that was most convenient for the participants. Prior to the commencement of interviews, the researchers obtained both oral and written informed consent from the participants, ensuring that they were fully aware of the study's purpose, procedures, and potential risks and benefits. The participants voluntarily agreed to participate and had the option to withdraw from the study at any point without facing consequences. The interviews with the students were conducted by the researchers themselves, who maintained a professional and unbiased stance throughout the process. Care was taken to establish a trusting and comfortable environment to encourage open and honest responses from the participants. The researchers had no dependent relationship with the students, minimizing the potential for bias and ensuring the integrity of the results obtained. The main question of the study was about the threats to preserving the dignity of the mother in maternity care. The interview process was based on the participants' initial answers, with probing questions such as “What do you mean?” or “Can you explain this more clearly?” being used to make the phenomenon clearer for researchers and participants. The individual interviews lasted between 30 and 90 minutes, with an average duration of 75 minutes. Interviews were conducted until saturation of collected data was achieved. All interviews were audio-recorded with the participant’s permission. After collating the data, we sent it back to the respondents to see if the data matched what they said. Any discrepancies in the interview results were discussed and timely corrected.

## Data analysis

Data analysis was performed using the MAXQDA software and the 8-step method proposed by Graneheim and Lundman^23^ to thoroughly examine the collected information. These steps were carefully followed to ensure a systematic and rigorous analysis process. First, immediately after each interview, we transcribed the entire interview verbatim, ensuring that every detail was accurately captured. From these transcripts, we identified meaningful segments known as “meaning units” and assigned primary codes to capture the essence of each unit. Next, we engaged in an inductive analysis approach. We examined the meaning units and primary codes, allowing themes and patterns to emerge organically from the raw data. This process led to the creation of classes that represented broader concepts within the dataset. To ensure consistency and validity, a sample of the text was independently coded by another member of our research team. This coding was then compared to the original coding, and any discrepancies were thoroughly discussed among the team members to reach a consensus. Throughout the analysis, the researcher responsible for coding regularly checked for agreement between the extracted codes, the perspectives of the participants, and the interpretations of the research team. This iterative process ensured the accuracy and reliability of the coding scheme. After coding the entire dataset, we conducted a stability check to assess the reliability of the coding. This involved revisiting the codes and their relationships to ensure their consistency and coherence across the data. Finally, we reviewed the codes, main classes, and sub-classes based on the entirety of the data. This comprehensive review allowed us to gain a holistic understanding of the dataset and the emerging themes within it.

To achieve bracketing, the researcher expressed and implemented the participants' experiences without any interference, attempting to set aside personal preconceptions and gain a new understanding of the subject. Prior to data collection, the researcher described their preconceptions about the threats to the dignity of mothers during labor and delivery.^24^

Guba and Lincoln's criteria, including the four criteria of credibility, dependability, confirmability, and transferability, were used to ensure the validity and strength of the qualitative data.^25^ *Credibility:* In this study, member and peer checks were used to ensure the accuracy of the data. Prolonged involvement with the phenomenon and engagement were also measures taken to increase the validity of the findings. *Dependability*: To check the reliability of the data obtained from the interviews, the supervisors and consultants reviewed and revised the implementation and coding. Three qualitative analysts coded and analyzed the data separately, and in 95% of cases, the results were similar to the analysis and coding of the research. *Confirmability:* The researcher clearly documented all the steps of the research so that other researchers can follow the data. Recording the statements of the participants, writing the statements, and reviewing them by the participants and expert supervisors helped provide criteria for verifying the research findings. *Transferability*: Participants were selected from different age groups and semesters to ensure maximum diversity. The researcher ensured the transferability of the study's findings by sharing them with a similar student population to the initial participants and gaining their endorsement. In addition, they further facilitated the possibility of replication by providing a thorough account of the participants' background, thereby enabling other researchers in a comparable setting to apply the findings.

## Ethical consideration

This study was part of a research project approved by the Ethics Committee of the Research Deputy at Kermanshah University of Medical Sciences in Kermanshah (approval code: IR. KUMS. REC.1401.085). We received permission to conduct the study from the School of Nursing and Midwifery as well as educational hospitals Emam Reza and Motazedi. Midwifery students were informed about the research objective and were made aware that participation was voluntary and that they could withdraw from the interview at any time. We assured them of the confidentiality of their information. Only those students who agreed to participate and provided written consent were interviewed

## Results

From the analysis of in-depth interviews, four themes encompassing sixteen sub-themes emerged, as shown in Table 1. These themes were formulated based on the perceptions of Iranian midwifery students about threats to maternal dignity during labor and birth. The identified themes included: *professional incompetence*, *abuse of power imbalance*, an exclusive focus on physical health with neglect of mental well-being, and *structural issues within the system.*

**Table 1.** The main themes and sub-themes extracted from Iranian midwifery students’ experiences regarding threats to maternal dignity during labor and childbirth.

Themes	Subthemes	The number of instances each subtheme came up during the interviews (n= 32)
Professional incompetence	Non-evidence-based interventions	21
	Normalization of disrespect and abuse	10
	Justifying violence in labor and delivery	19
Abuse of power imbalance	The commanding tone during the care	13
	Humiliation of the mother	22
	Beating the mother	18
	Discrimination	11
	Masterful expectations from the mother	4
Caring only for physical and not mental health	Preferring the use of pharmaceutical pain relief methods vs non-pharmacological	3
	Reactions only to changes in maternal and fetal physical signs	5
	Ignoring maternal suffering, anxiety, and stress	14
	Ignoring the need for the presence of a companion or spouse	25
Structural issues within the system	High workload and fatigue among staff	28
	Inappropriate environment	26
	Ignoring dignity by policies, processes and laws	14
	Facilitation of disrespect and abuse in teaching hospitals	21

## Theme 1. Professional incompetence

Professional incompetence reflected non-evidence-based interventions, the normalization of disrespect and abuse by healthcare providers, and the justification of violence during labor and delivery. Midwifery students expressed the professional incompetence of health providers as the primary threat to maternal dignity during labor and childbirth. Professional incompetence was identified as having three aspects: non-evidence-based interventions, the normalization of disrespect and abuse, and the justification of violence during labor and delivery.

### Non-evidence-based interventions

Many midwifery students, particularly those close to graduation, believed that midwives not being up-to-date and not working scientifically was a serious threat to neglecting the mother's dignity. The midwifery students stated that manipulating the mother without paying attention to evidence-based medicine was distressing for the mother and could endanger the lives of both the mother and the fetus. A student said:

“Unnecessary interventions and vaginal examinations are annoying for the mother. Midwives must know the updated knowledge, they can control childbirth well, and don't manipulate the mother so much.” (Participant 7,)

Despite the fact that the following observation comes from a different student, it reinforces the concerns raised about the quality of care in labor and delivery settings. This student reported witnessing instances where established protocols were not followed during the induction process, resulting in an excessive number of inductions being performed. Furthermore, the student noted that they had observed numerous instances where unnecessary episiotomy incisions were made during delivery, even though there was no medical indication for such a procedure. This highlights a troubling pattern of deviation from evidence-based practices and an overreliance on potentially harmful interventions. Such behavior not only undermines the principles of patient-centered care but also exposes mothers and their babies to avoidable risks and complications.

“Induction is not done according to what the protocol says, and they do more inductions. Many times, I have seen that, for example, in a delivery, an episiotomy incision was made that did not need to be made at all, or similar events.” (Participant 2).

The midwifery students elaborated on the importance of adhering to evidence-based medicine in the context of childbirth, emphasizing the value of facilitating a physiological labor process for the mother. Supporting the mother in experiencing the natural progression of labor and delivery is a prime example of evidence-based medicine in practice according to them. This approach not only minimizes the need for unnecessary interventions but also contributes to reducing the mother's pain and discomfort throughout the process.

“It is preferable to let the mother experience the natural process of childbirth, as this approach minimizes interventions and reduces the mother's pain and discomfort” (Participant 1).

By prioritizing the natural course of childbirth, healthcare providers can ensure that mothers receive care that is in line with best practices and respects the physiological processes involved.

### Normalization of disrespect and abuse

For certain healthcare providers, exhibiting disrespect towards pregnant women has become normalized, to the point where they feel comfortable insulting them even in the presence of their superiors. These individuals often speak openly about their inappropriate behavior towards mothers in labor. A midwifery student stated:

“Instead of respecting mothers, disrespect has regrettably become commonplace. For some staff members, demeaning pregnant women has become the norm, which is concerning. Over time, this lack of respect can evolve into a deep-rooted culture, causing new staff members and students to embrace this detrimental behavior” (Participant 7).

This troubling phenomenon, wherein certain staff members had routinely demeaned and belittled pregnant women, raised significant concerns among midwifery students. Over time, this lack of respect could become entrenched within the organizational culture, influencing both new staff members and students to adopt these harmful behaviors. It was crucial to address this issue and promote a culture of empathy and understanding to ensure the well-being of mothers and their newborns.

### Justifying violence in labor and delivery

Several midwifery students had identified a concerning factor that posed a risk to pregnant women: the occurrence of violence against mothers during childbirth. In some cases, this violence was mistakenly justified as a means to protect the health and well-being of both the mother and her unborn child. This misguided notion might have stemmed from a lack of understanding or adherence to evidence-based practices, perpetuating a harmful culture within healthcare settings.

“Sometimes midwives argue with the mother, saying, ‘Ma'am, open your legs, your baby is dying, suffocating, etc.,' essentially threatening her with the most horrifying thing a mother can experience – the death of a baby she has carried for nine months. When asked why they disrespected the mother, they justify it as being for the sake of her health and that of her child.” (Participant 6)

They underscored the importance and heightened awareness of the detrimental effects of such actions, advocating the prioritization of evidence-based, compassionate care to ensure the safety and well-being of mothers and their newborns throughout the childbirth process.

## Theme 2. Abuse of power imbalance

The abuse of power imbalance was manifested through five critical sub-themes. Firstly, the commanding tone during care referred to an authoritarian relationship and a deficiency in informed consent procedures. Secondly, the humiliation of the mother emphasized how the actions of midwives, gynecologists, and other staff could instigate maternal frustration and plummeting self-esteem. The third aspect was physical violence against the mother, including instances of beating during labor and childbirth. The fourth, discrimination, outlined the prejudiced treatment that mothers often faced. Lastly, masterful expectations from the mother were noted, indicating the imposition of unreasonable demands and pressures on mothers during the birthing process. Each of these sub-themes was explored in detail to fully understand their implications and consequences.

### The commanding tone during the care

A number of students have observed that the way some midwives interact with mothers during labor is reminiscent of a master-servant dynamic, reflecting a lack of respect for the dignity and autonomy of the mothers. One student poignantly described this issue by stating, “Certain midwives appear to adopt an authoritative role, treating mothers as if they were subordinates.” They issue directives and communicate in a manner that undermines the mothers' dignity and autonomy.

“Some midwives act as if they are the boss and mothers are their servants. They issue commands as though they are speaking to a subordinate, so that one of the midwives once said to the mother: Change your clothes and clean your underwear and put it in the trash can. You ruined my mood.” (Participants 22)

This highlights the need to promote a culture of empathy, respect, and collaboration within healthcare settings to ensure positive experiences for mothers during childbirth.

### Humiliation of the mother

The midwifery students expressed concerns about the negative impact of demeaning behavior towards mothers during labor, highlighting how such treatment can erode their dignity and self-esteem. One student shared a poignant example, stating:

“When a mother complains about too many examinations or anything else that bothers her, we quickly remind her that she chose to come to the teaching hospital! Or we question her knowledge of labor and delivery. In my opinion, this is very harmful; it undermines the mother's dignity and self-esteem, making her feel ashamed or foolish.” (Participant 1).

This underscores the importance of fostering a compassionate and supportive environment during childbirth to ensure positive experiences for mothers.

### Beating the mother

An additional concern that emerged is the occurrence of physical or other forms of violence during labor and delivery, which severely undermines a mother's dignity and poses a substantial threat to her sense of self-worth. Within this context, a student shared a relevant experience, as follows:

I even remember during a delivery, the midwife responsible for the birth repeatedly hit the mother's feet, causing her to cry. A midwife has no right to strike a mother for any reason. She is also a human being. We must respect her, even if she screams. (Participant 30)

### Discrimination

Midwifery students emphasized that regardless of a patient's background, culture, or beliefs, mothers should be respected, listened to, and have their questions answered. The students highlighted that services should be provided equally to everyone, irrespective of their social class or drug use, as discrimination can threaten a mother's dignity. A midwifery student stated that regardless of the patient's background, culture, or beliefs, we should respect the mother, listen to her, and answer any questions she may have.

Sometimes, we see that mothers from higher social classes receive better treatment, while those from lower socioeconomic backgrounds or who struggle with drug abuse are disregarded or, even worse, disrespected. (Participant 9)

Many students believed that recommended patients receive much better services than others and that discrimination between patients was a serious threat to the maintenance of human dignity. One of them said:

I once saw a woman in a private room, they [midwifes] treated her very respectfully and kindly, and they injected her with painkillers. Later I found out that she was recommended by the governor`s family. In fact, it is very disturbing that midwives do not treat mothers in labor the same way. In fact, it is very worrying that midwives do not treat mothers during labor equally. (Participant 20)

Additionally, some students observed that certain mothers may not receive respectful maternity care because they have multiple children or were married at a young age. For example, midwives or gynecologists might interact with pregnant mothers in a harsh and insensitive manner, employing a negative tone and making derogatory comments. One student recounted overhearing a midwife say:

You were in such a rush to get married at a young age. You're still wet behind the ears! (Participant 28)

### Masterful expectations from the mother

In this section, the students described their experience of how the staff at the hospitals expected the mothers to be. They expected a mother who comes to the hospital to possess comprehensive knowledge about everything from the start. This is unreasonable to demand that she quickly complete tasks and blindly follow instructions the students expressed. Instead, we should consider the mother's preferences, identify what is easier and better for her, and provide education without neglect. One student observed an incident where a midwife scolded a mother, saying:

“What do you mean, you don't know how to say hi?” The midwife spoke in an angry tone and chastised the mother for not greeting her correctly upon entering. This treatment shocked the mother and diminished her self-esteem. The midwife's expectations were unreasonable, causing the mother unnecessary stress. (Participant 1)

## Theme 3. Caring only for physical and not mental health

Another prominent compromise to a mother’s dignity was identified as the undue focus on physical health, often sidelining mental well-being. This issue was divided into four subcategories: a preference for pharmaceutical pain relief methods over non-pharmacological ones, responses limited solely to changes in maternal and fetal physical indicators, a disregard for maternal suffering, anxiety, and stress, and an oversight of the necessity for a companion or spouse's presence. The majority of midwifery students had reported that midwifery care was primarily targeting the physical aspects of a mother's health, while largely neglecting her mental well-being.

### Preferring the use of pharmaceutical pain relief methods vs non-pharmacological

One instance of disregarding a mother’s honor and dignity during labor and delivery, as reported by midwifery students, is the preference for pharmaceutical pain relief methods over non-pharmacological alternatives.

There is no doubt that mothers experience pain during childbirth, but it is important to utilize non-pharmacological methods to alleviate the pain while also addressing the mother's support needs. Employing these methods is the mother's right, whereas opting for pharmaceutical methods often caters to the convenience of the midwife who may not want to stay with the mother. (Participant 25)

One example of disregarding the honor and dignity of the mother during labor and delivery, as reported by midwifery students, was the preference for pharmaceutical pain relief methods over non-pharmacological alternatives.

### Reactions only to changes in maternal and fetal physical signs

The statements from some midwifery students in this study suggested that the threat to a mother's dignity was associated to service providers focusing solely on the physical issues of the mother and fetus during labor and delivery.

Midwives and gynecologists primarily provide care in response to physical health concerns for the mother and fetus, such as routinely monitoring the mother's vital signs, bleeding, amniotic sac rupture, and fetal heart rate. […] a mother is sitting there, stressed and wondering about the progress of her labor, […] she received no information, education, or care. This approach significantly undermined the mother's dignity. (Participant 3)

### Ignoring maternal suffering, anxiety, and stress

According to students' perceptions, neglecting maternal suffering, anxiety, and stress poses a significant threat to the dignity of mothers, which can impact the quality of a woman's future life.

Pregnancy and childbirth are not solely about maintaining the physical safety of women; we must also have a plan to make childbirth a pleasant experience and create positive memories for the mother. Mothers endure suffering, stress, and anxiety during childbirth. Midwives should alleviate the mother's stress and anxiety by providing information, obtaining informed consent, seeking permission before performing examinations, respecting the mother's opinion, and preserving her dignity. Women's memories, such as pregnancy and childbirth experiences, can last a lifetime and potentially affect their quality of life. (Participant 5)

### Ignoring the need for the presence of a companion or spouse

Based on the perspectives of the majority of participants, the prohibition of a companion or spouse's presence during labor and delivery had been damaging to the mother's dignity. Some mothers had requested their husbands to be with them, but regrettably, the staff frequently disregarded these requests. One of the students expressed this concern, stating.

Regrettably, when mothers enter labor and delivery, they become entirely isolated from the outside world. This means the mother cannot use the phone, has no companion, and her husband cannot be with her. Once, a woman was crying, pleading to see her husband and have him by her side, but the midwife paid no attention to her request. These actions pose serious threats to the mother's dignity. (Participant 4)

## Theme 4. Structural issues within the system

Structural problems of the system reflected high workload and fatigue among staff, inappropriate environments, a lack of dignity in policies, processes, and laws, and the facilitation of disrespect and abuse in teaching hospitals. This category included four subcategories: high workload and staff fatigue, inappropriate environment, ignoring dignity by policies, processes and laws, and facilitation of disrespect and abuse in teaching hospitals.

***High workload and fatigue among staff*** Midwifery students expressed concerns about the impact of staffing shortages and excessive workloads on caregivers, noting that these elements can contribute to fatigue and, in turn, affect the quality of performance and respectful maternity care provided.

One student elaborated on this issue, stating:

There are instances when midwives experience exhaustion due to long night shifts and overwhelming workloads, which can create challenging circumstances during labor. As a result, they may be under significant psychological pressure and stress. A caregiver burdened by such stress is less likely to provide the level of attention and care that the patient truly deserves. (Participant 32)

This highlights the importance of addressing workforce-related issues in maternity care settings to ensure that both mothers and their newborns receive the optimal level of care and support during childbirth.

### Inappropriate environment

Midwifery students believed that providing a comfortable, clean, and quiet labor and birth environment with sufficient equipment is essential for preserving the dignity of mothers. On the contrary, an inappropriate environment is a threat to the dignity of mothers.

Even the clothes given to a patient are not really beautiful. Neither the clothes nor the environment is something that makes a person have a good memory. (Participant 14)

The students conveys that the space being is unsatisfactory and uncomfortable for the mother. The elements mentioned, such as the colors, beds, and overall conditions of the room, contribute to an environment that does not promote relaxation or a sense of peace for mothers. The students seemed to be concerned for the mother's well-being in the setting.

The space is not good, and it does not give the mother peace. Neither the colors used in the environment, nor the beds, nor the conditions of the room are really such that the mother feels relaxed. (Participant 1)

### Ignoring dignity by policies, processes, and laws

One of the participants stated that ignoring the preservation of mothers' dignity by policies, processes, and even laws is a serious threat. She believes that when the preservation of the mother's dignity is not considered in the system, it can be concluded that the system does not prioritize it. She also adds:

‘Those who behave more appropriately with the patient should be encouraged, and midwives who value the dignity of the mother should be appreciated.' (Participant 16).

### Facilitation of disrespect and abuse in teaching hospitals

Most midwifery students believed that university hospitals, without taking measures to protect the dignity of the mother, pose a serious threat. They strongly condemn the invasive practice of allowing multiple individuals, including midwifery students, instructors, and residents, to perform vaginal examinations on a mother during childbirth. The students argued that this practice is highly inappropriate and compromises the mother's dignity. They believe that this system makes the teaching hospital an unsuitable place for childbirth due to the lack of respect for the mother's privacy and personal boundaries.

While it is true that teaching hospitals are necessary for students to learn, it is unacceptable for everyone who passes by to perform a vaginal examination. The mother should not be asked to keep her legs open so that the midwifery student, their instructor, intern, and the 1st, 2nd, 3rd-year residents can examine her vagina. This is highly inappropriate. I feel that it is not a suitable place for childbirth, and the mother's dignity is compromised in this system. (Participant 21)

Furthermore, these facilities frequently have insufficient or outdated equipment, which could impact the quality of care provided. A high number of students each perform vaginal examinations on the mother in a repetitive manner, causing distress, and discomfort. The overall impression conveyed is that teaching hospitals may not provide the best environment for mothers due to the lack of proper equipment and the intrusive nature of repeated examinations by multiple students.

Teaching hospitals often lack the necessary equipment, and when they do have it, it tends to be old and outdated. Additionally, there is an excessive number of students, each of whom bothers the mother by performing vaginal examinations one after another. (Participant 8)

## Discussion

The aim of this study was to explore the experiences of midwifery students regarding threats to women's dignity during childbirth. The participating students identified factors such as professional incompetence, abuse of power imbalance, caring only for physical and not mental health, and structural issues within the system as threats to a mother's dignity during childbirth. Our research indicated that insufficient midwifery professional competence is a significant threat to maternal dignity. A lack of awareness regarding professional ethics and outdated care practices results in non-evidence-based interventions, the normalization of disrespect, and the justification of violence during childbirth as routine care.

The findings of a Cochrane study revealed that harm to mothers increases due to a lack of accurate knowledge and professional competence.^26^ Healthcare providers have ethical, legal, and professional obligations to offer safe and respectful care.^12^ Preserving patients' dignity is one of the most crucial professional and ethical responsibilities of healthcare professionals, and all caregivers are expected to respect their patients' dignity. When care providers are up to date, both mother's satisfaction and the quality of care provided improve. However, in Iran, maternity services primarily focus on medical interventions rather than midwifery care. Procedures such as episiotomy for first-time mothers and the use of oxytocin during labor are considered routine in Iran without obtaining women's informed consent or involving them in the decision-making process. In contrast, in European countries, less than half of women receive oxytocin or episiotomy during labor, and the routine use of these procedures without the woman's permission is deemed obstetric violence.^20^

Midwifery students observed that midwives frequently used a commanding and racial tone when interacting with mothers, which contributes to childbirth trauma. This is in line with previous studies conducted in South Africa, Netherland, and Ghana where midwifery students witnessed disrespectful care.^27,28^ This approach is not in line with global standards for woman-centered maternity services. Factors such as a commanding tone, humiliation of the mother, physical violence, discrimination, and unrealistic expectations during childbirth all threaten women's dignity during labor and delivery. However, raising awareness through group reflective activities can help students recognize and identify this type of violence, which is often perceived as in obstetric during the childbirth.^29^ Similar studies have reported disrespectful and abusive behavior by staff, including discrimination, lack of privacy, absence of informed consent, and physical abuse in maternity centers.^7,20,21,22^ Globally, it's a common occurrence for women to face demeaning and disrespectful behavior during their prenatal and childbirth care. The prevalent disregard for a mother's dignity during childbirth, which is a consequence of abusive and disrespectful conduct by service providers, is a cause for widespread concern.^30^

Many midwifery students pointed out that current care measures prioritize preventing maternal and neonatal mortality and morbidity, often neglecting the psychological well-being of mothers. The preference for pharmaceutical pain relief methods over non-pharmacological alternatives, responding solely to changes in maternal and fetal physical signs, disregarding maternal suffering, anxiety, and stress, and overlooking the need for a companion or spouse all indicate this neglect. Woman-centered care must encompass measures to ensure both physical and mental safety. The World Health Organization recommends supporting and respecting mothers during labor and delivery to alleviate their fear and anxiety.^31^ Mothers who desire a vaginal birth require emotional and psychological support from midwives and nurses.^32^ Overlooking patients' psychological needs puts their dignity at risk. Even though non-pharmacological pain relief methods can help manage pain, the psychological support needed by mothers during childbirth remains crucial.^32,33^ The integration of trained companions or partners into the birthing team is an indispensable aspect of preserving women's dignity during labor and childbirth. Such support offers reassurance and comfort, thereby enhancing the physical and mental well-being of the mother.

However, in Iranian government hospitals, the exclusion of husbands from delivery rooms serves as a violation of this support system. The lack of regard for women's expressed desires and informed consent in such decisions is a manifestation of obstetric violence.^10^

Midwifery students' accounts of threats to dignity frequently mentioned structural system issues. These problems encompassed high workload and fatigue among staff, unsuitable environments, disregard for dignity by policies, processes, and laws, and the facilitation of disrespect and abuse in teaching hospitals. Women in the delivery room should receive care in an environment where healthcare workers promote their dignity by maintaining privacy, providing attentive care, and ensuring that patients' preferences regarding care and treatment are respected.^34^ In such an atmosphere, women's dignity is preserved, and favorable outcomes are achieved.^34,35^ The results of one study corroborated our findings, demonstrating that fatigue caused by long shifts can negatively affect staff's medical decisions, memory, mood, and behavior, thereby undermining mothers' dignity.^36^ However, another study contradicted this finding, asserting that respectful behavior does not fundamentally depend on the availability of funds and shift work beyond improving the health system to provide safe conditions for childbirth.^24^ In any situation, respect largely relies on the goodwill, professionalism, and commitment of individuals within the system.^12,25^

Currently, in Iran, most midwives, like other sectors of society, are dissatisfied with their job status, and low wages and salaries are cited as the most significant concerns.^37^ Considering the numerous services midwives provide in the birthing unit in Iran, and despite the fact that they perform more than 70% of all vaginal births in the country, their salaries and benefits do not adequately reflect the challenges of their work.^38^

Besides these systemic issues, some midwifery students believed that disrespect and abuse were enabled in teaching hospitals. Despite efforts to uphold the dignity of pregnant mothers, their preservation of dignity and human rights in educational hospitals is compromised. To achieve this vital goal of respect for mothers, it is essential to not only teach ethics to students at various levels of medical sciences but also to revise hospital structures and regulations.^38^

This qualitative study was the first of its kind, conducted on midwifery students in Iran. The strength of this study lies in interviewing an adequate number of midwifery students. Although the study was conducted on midwifery students in Kermanshah, the results may be applicable to other provinces in Iran, as well as similar contexts and cultures in other low- and middle-income countries. By clarifying the views of midwifery students on the threats to mothers’ dignity, we can improve the quality of professional ethics training and examine the challenges of preserving mothers' dignity during labor and childbirth.

## Limitations of the study

For this study, we selected only midwifery students as participants. In future research, different populations such as medical students and obstetricians could be included to investigate threats to women's dignity during childbirth from a broader perspective. It’s important to note that our study participants experienced birth care in teaching hospitals where obstetricians manage all vaginal births, and midwives have a more limited role in natural births. This factor may restrict the generalizability of the results to all Iranian hospitals.

## Conclusion

This research underscores that mothers' dignity is at risk due to unprofessional conduct among midwives, inconsistent use of evidence-based medicine, and systemic flaws within healthcare settings. Our findings illuminate the urgent need for policies and programs that address these issues in a comprehensive manner. Central to this is the development and application of dignity-focused guidelines in midwifery services, paired with professional education emphasizing respect for mothers' dignity and evidence-based practices. Moreover, this study encourages adopting the World Health Organization's five recommended actions for dignity during facility-based childbirth. Implementing these steps may lead to improved maternal healthcare, increased patient satisfaction, and a more respectful culture of care globally.

## References

[1] Healy S Humphreys E Kennedy C . Can maternity care move beyond risk? Implications for midwifery as a profession. Br J Midwifery 2016; 24: 203–209.

[2] Leal Mdo C Gama SGNda Pereira APE , et al. The color of pain: racial iniquities in prenatal care and childbirth in Brazil. Cad Saúde Pública 2017; 33: e00078816. 10.1590/0102-311X00078816 28746555

[3] Smith-Oka V Rubin SE Dixon LZ . Obstetric violence in their own words: how women in Mexico and South Africa expect, experience, and respond to violence. Violence Against Women 2022; 28: 2700–2721. 34766519 10.1177/10778012211037375

[4] Bohren MA Vogel JP Hunter EC , et al. The mistreatment of women during childbirth in health facilities globally: a mixed-methods systematic review. PLoS Med 2015; 12: e1001847. 26126110 10.1371/journal.pmed.1001847 PMC4488322

[5] Halperin O Goldblatt H Noble A , et al. Stressful childbirth situations: a qualitative study of midwives. J Midwifery Wom Health 2011; 56: 388–394. 10.1111/j.1542-2011.2011.00030.x 21733111

[6] Jardim DMB Modena CM . Obstetric violence in the daily routine of care and its characteristics. Rev Lat Am Enfermagem 2018; 26: e3069. 30517571 10.1590/1518-8345.2450.3069 PMC6280177

[7] Sadler M Santos MJ Ruiz-Berdún D , et al. Moving beyond disrespect and abuse: addressing the structural dimensions of obstetric violence. Reprod Health Matters 2016; 24: 47–55. 27578338 10.1016/j.rhm.2016.04.002

[8] Shabot SC . We birth with others: towards a beauvoirian understanding of obstetric violence. Eur J Wom Stud 2021; 28: 213–228.

[9] Davis-Floyd R Premkumar A . Obstetric violence and systemic disparities: can obstetrics Be humanized and decolonized? Berghahn Books, 2023.

[10] Sen G Reddy B Iyer A . Beyond measurement: the drivers of disrespect and abuse in obstetric care. Reprod Health Matters 2018; 26: 6–18. 30189791 10.1080/09688080.2018.1508173

[11] Chadwick RJ . Obstetric violence in South Africa. SAMJ: S Afr Med J 2016; 106: 423–424. 10.7196/SAMJ.2016.v106i5.10708 27138653

[12] Beauchamp TL Childress JF . Principles of biomedical ethics. 7th ed. Oxford: Oxford University Press, 2009.

[13] World Medical Association . World medical association declaration of Helsinki: ethical principles for medical research involving human subjects. JAMA 2013; 2013: 2191–2194. 10.1001/jama.2013.281053 24141714

[14] Duggan PS Geller G Cooper LA , et al. The moral nature of patient-centeredness: is it “just the right thing to do”? Patient Educ Counsel 2006; 62: 271–276. 10.1016/j.pec.2005.08.001 16356677

[15] Sensen O . Kant on human dignity. Berlin: Walter de Gruyter, 2011.

[16] Sandman L Munthe C . Shared decision-making and patient autonomy. Theor Med Bioeth 2009; 30: 289–310. 19701695 10.1007/s11017-009-9114-4

[17] Stoljar N . Informed consent and relational conceptions of autonomy. J Med Philos 2011; 36: 375–384. 21825176 10.1093/jmp/jhr029

[18] Gallagher A Wainwright P Baillie L , et al. The RCN dignity survey: implications for leaders: ann gallagher and colleagues highlight the significant role played by nurse leaders in developing and maintaining a culture of dignity for patients and staff in healthcare services. Nurs Manag 2009; 16: 12–17. 10.7748/nm2009.07.16.4.12.c7132 19639904

[19] Eri TS Berg M Dahl B , et al. Models for midwifery care: a mapping review. Eur J Midwifery 2020; 4: 30. 33537631 10.18332/ejm/124110 PMC7839165

[20] Malatji R Madiba S . Disrespect and abuse experienced by women during childbirth in midwife-led obstetric units in Tshwane district, South Africa: a qualitative study. Int J Environ Res Publ Health 2020; 17: 3667. 10.3390/ijerph17103667 PMC7277802 32456063

[21] Abuya T Warren CE Miller N , et al. Exploring the prevalence of disrespect and abuse during childbirth in Kenya. PLoS One 2015; 10: e0123606. 25884566 10.1371/journal.pone.0123606 PMC4401776

[22] Sando D Ratcliffe H McDonald K , et al. The prevalence of disrespect and abuse during facility-based childbirth in urban Tanzania. BMC Pregnancy Childbirth 2016; 16: 236. 27543002 10.1186/s12884-016-1019-4 PMC4992239

[23] Graneheim UH Lundman B . Qualitative content analysis in nursing research: concepts, procedures and measures to achieve trustworthiness. Nurse Educ Today 2004; 24: 105–112. 14769454 10.1016/j.nedt.2003.10.001

[24] Paturzo M Petruzzo A Bertò L , et al. The lived experience of adults with heart failure: a phenomenological study. Ann Ig 2016; 28: 263–273. 27479762 10.7416/ai.2016.2105

[25] Guba EG Lincoln YS . Guidelines and checklist for constructivist (aka fourth generation) evaluation. Sage publication, 2001, p. 15.

[26] Mehretie Adinew Y Kelly J Marshall A , et al. Care providers’ perspectives on disrespect and abuse of women during facility-based childbirth in Ethiopia: a qualitative study. Int J Wom Health 2021; 13: 1181–1195. 10.2147/IJWH.S333863 PMC8643202 34876861

[27] van der Waal R Mitchell V van Nistelrooij I , et al. Obstetric violence within students’ rite of passage: the reproduction of the obstetric subject and its racialised (m)other. Agenda 2021; 35: 36–53.

[28] Moyer CA Rominski S Nakua EK , et al. Exposure to disrespectful patient care during training: data from midwifery students at 15 midwifery schools in Ghana. Midwifery 2016; 41: 39–44. 27522042 10.1016/j.midw.2016.07.009

[29] Mena-Tudela D González-Chordá VM Soriano-Vidal FJ , et al. Changes in health sciences students’ perception of obstetric violence after an educational intervention. Nurse Educ Today 2020; 88: 104364. 32120084 10.1016/j.nedt.2020.104364

[30] Bowser D Hill K . Exploring evidence for disrespect and abuse in facility-based childbirth, 2010.

[31] American College of Nurse-Midwives. Midwives Alliance of North America, National Association of Certified Professional Midwives . Supporting healthy and normal physiologic childbirth: a consensus statement by the American college of nurse-midwives, midwives alliance of North America, and the national association of certified professional midwives. J Midwifery Wom Health 2012; 57: 529–532. 10.1111/j.1542-2011.2012.00218.x 22954092

[32] Hodnett ED Gates S Hofmeyr GJ , et al. Continuous support for women during childbirth. Cochrane Database Syst Rev. Epub ahead of print. DOI: 10.1002/14651858.CD003766.pub5. 12917986

[33] Karlström A Nystedt A Hildingsson I . The meaning of a very positive birth experience: focus groups discussions with women. BMC Pregnancy Childbirth 2015; 15: 251. 26453022 10.1186/s12884-015-0683-0 PMC4600272

[34] Organization WH . WHO recommendations intrapartum care for a positive childbirth experience. Geneva: World Health Organization, 2018. 30070803

[35] Berger BO Strobino DM Mehrtash H , et al. Development of measures for assessing mistreatment of women during facility-based childbirth based on labour observations. BMJ Glob Health 2022; 5: e004080. 10.1136/bmjgh-2020-004080 PMC8353173 34362791

[36] Jasti H Hanusa BH Switzer GE , et al. Residents’ perceptions of a night float system. BMC Med Educ 2009; 9: 52. 19650924 10.1186/1472-6920-9-52 PMC2728710

[37] Moridi M Pazandeh F Hajian S , et al. Midwives’ perspectives of respectful maternity care during childbirth: A qualitative study. PLoS One 2020; 15: e0229941. 32150593 10.1371/journal.pone.0229941 PMC7062245

[38] Hosseini FA Momennasab M Yektatalab S , et al. Patients’ perception of dignity in Iranian general hospital settings. Nurs Ethics 2019; 26: 1777–1790. 29734885 10.1177/0969733018772078

